# Expression of *15-PGDH* Regulates Body Weight and Body Size by Targeting JH in Honeybees (*Apis mellifera*)

**DOI:** 10.3390/life15081230

**Published:** 2025-08-03

**Authors:** Xinying Qu, Xinru Zhang, Hanbing Lu, Lingjun Xin, Ran Liu, Xiao Chen

**Affiliations:** 1 State Key Laboratory of Resource Insects, Institute of Apicultural Research, Chinese Academy of Agricultural Sciences, Beijing 100193, China; 82101235489@caas.cn (X.Q.); luhanbing1254@163.com (H.L.); 2 College of Bioscience and Resource Environment, Beijing University of Agriculture, Beijing 102206, China; 202330112016@bua.edu.cn (X.Z.); xinlingjun@bua.edu.cn (L.X.); 3 Beijing Tianbaokang Hi-Tech Development Co., Ltd., Beijing 100193, China; liuran-job@sohu.com

**Keywords:** *15-PGDH*, JH, emergence weight, morphometric features, RNAi

## Abstract

Honeybees (*Apis mellifera*) are pollinators for most crops in nature and a core species for the production of bee products. Body size and body weight are crucial breeding traits, as colonies possessing individuals with large body weight tend to be healthier and exhibit high productivity. In this study, small interfering RNA (siRNA) targeting *15-Hydroxyprostaglandin dehydrogenase* (*15-PGDH*) was incorporated into the feed for feeding worker bee larvae, thereby achieving the silencing of this gene’s expression. The research further analyzed the impact of the RNA expression level of the *15-PGDH* gene on the juvenile hormone (JH) titer and its subsequent effects on the body weight and size of worker bees. The results show that inhibiting the expression of *15-PGDH* in larvae could significantly increase JH titer, which in turn led to an increase in the body weight of worker bees (1.13-fold higher than that of the control group reared under normal conditions (CK group); *p* < 0.01; SE: 7.85) and a significant extension in femur (1.08-fold longer than that of the CK group; *p* < 0.01; SE: 0.18). This study confirms that *15-PGDH* can serve as a molecular marker related to body weight and size in honey bees, providing an important basis for molecular marker-assisted selection in honey bee breeding.

## 1. Introduction

Honeybees are social insects and are incredibly important pollinators worldwide [1,2]. Most crops in the world rely on insect pollination to produce seeds and fruits. With their unique morphological structures and biological characteristics, honeybees are regarded as the best pollinators to promote high yield and quality of crops such as grains, vegetables, fruits and oilseeds [3,4]. Also, honeybees provide several bee products that are beneficial to human health.

High body weight and size are important goals in breeding work in honeybees. Studies report that worker bees with larger body size typically have longer proboscises and stronger flight muscles, which can expand their foraging range [5]. Larger individuals have better stress resistance and can better maintain sufficient body temperature in cold environments [6]. The larval stage is a critical period for the growth and development of honeybees, and their body size is mainly determined by this stage [7]. Numerous factors influence the development of honeybee larvae, including nutrition [8], temperature [9], hormones [10], DNA methylation [11,12] and genes [13,14]. It is found that juvenile hormone (JH) plays a pivotal role in the larval development in honeybees [15]. JH is synthesized and secreted by the *corpora allata* and subsequently released into the hemolymph. It plays an important role in regulating growth, organic development, metamorphosis and reproduction in insects, standing as one of the most crucial classes of insect hormones [16,17,18,19]. It is found that sustained high JH titer prolongs the larval developmental phase, leading to larger body weight and size [20]. Also, JH promotes ovarian weight gain and increases the number of ovarioles in queens [21]. In honey bee development, queen larvae ovaries develop through cell proliferation, while worker larvae ovaries undergo programmed cell death, resulting in reproductive capacity loss [22]. Therefore, it can be inferred that if JH titer in worker larvae is significantly increased during the larval development, it may redirect the worker larval development toward the queen way, resulting in workers with a larger body size. JH titers are regulated by multiple internal and external factors [23]. Recent progress has elucidated the regulatory interplay between JH and gene expression. Studies have found that ame-miR-5119 negatively regulates the expression of *ecdysis-triggering hormone (Eth)*, indirectly inhibits the expression of *Eth receptor* (*Ethr*), JH acid methyltransferase gene (*Jhamt*) and kruppel homolog 1 gene (*Kr-h1*), and affects the JH biosynthesis, thereby preventing the metamorphic transition from larva to pupa in worker bees [24]. Wang et al. [25] applied RNAi technology and found that *AmIlp-1* can regulate the synthesis of JH in the bee brain and ovarian development. Based on the differential expression of *AmIlp-1* and *AmIlp-2* between queens and workers, as well as the regulatory effects of *AmIlp-1* on JH and ovaries, it can be inferred that *AmIlp-1* and *AmIlp-2* were associated with the larval developmental process in honeybees.

*15-Hydroxyprostaglandin dehydrogenase* (*15-PGDH*) serves as the sole catabolic enzyme for prostaglandin E2 (PGE2). It catalyzes the conversion of PGE2 into 15-keto-PGE2, which exhibits significantly reduced biological activity. Consequently, *15-PGDH* is recognized as the primary negative regulator of PGE2 [26]. PGE2 mediates its effects through G protein-coupled receptors EP1-4, activating signaling pathways including PKA/CREB, GSK3β/β-catenin, PI3K/AKT/mTOR and NF-κB, thereby exerting diverse biological functions [27,28]. Yan et al. [29] revealed a key finding through comparative analysis of *15-PGDH* expression levels in normal colon tissues and colon cancer tissues: the expression level of *15-PGDH* in colon cancer tissues was significantly reduced by at least 17-fold compared with that in normal colon tissues. Further studies showed that when the expression of *15-PGDH* in colon cancer tissues was enhanced by external means, the growth of these cancer cells was significantly inhibited. It has also been found in experimental models of liver cancer cells that increasing the expression level of *15-PGDH* can effectively trigger the apoptosis process of tumor cells, thus revealing its potential role in promoting the death of liver cancer cells [30]. In our previous study, transcriptome sequencing was conducted to identify the differently expressed genes during the larval developmental stages between queens and workers. It was found that the expression of *15-PGDH* in workers gradually increased during the larval stage, and its expression was significantly higher in workers compared to queens. Based on these findings, we hypothesize that *15-PGDH* may be a candidate gene influencing honeybee larval development.

*15-PGDH* is an enzymatic protein, chemically classified as a short-chain dehydrogenase. It catalyzes the reaction between hydroxyl groups (-OH) and hydrogen atoms (H), acting as a catalyst to facilitate the reaction process. Since the precursor of JH synthesis is an acid, it is hypothesized that the high expression of *15-PGDH* may inhibit the JH synthesis process, thereby reducing low JH titers and ultimately influencing the larval development in honeybees. Based on our previous research on larval development (unpublished), this study aims to elucidate whether *15-PGDH* expression affects JH titer and subsequently influences honeybee larval development.

## 2. Materials and Methods

### 2.1. Ethics Statement

The honeybee colonies used in this study were maintained by the Institute of Apicultural Research, Chinese Academy of Agricultural Sciences (IAR, CAAS), Beijing, China (116°11′57″ E, 40°0′23″ N). The ethics committee of the institute approved the experimental protocol (Approval No.: MFSDWLLSC-2024-07; approval date: 6 August 2024). 

### 2.2. Sampling

All samples were obtained from *Apis mellifera ligustica* honeybee colonies. In June 2024, honey bee colonies were reared using standard beekeeping techniques. To obtain larvae of the same age, the queen was caged to restrict it from laying eggs on a specific comb for 12 h. On the fourth day after queen caging, the larvae were grafted from the brood comb into a 48-well culture plate. This time was defined as 0 h. Then, the larvae were reared in the laboratory following the method described by Schmehl et al. [31]. The food mainly included royal jelly, glucose, fructose and yeast extract. The dietary composition was slightly adjusted daily based on the larval stage, following the established formulations detailed by Schmehl et al. [31] (Table S1). At this time, the larvae were divided into three groups, including the experimental group supplemented with siRNA (*15-PGDH* siRNA group), the negative control group (nonsense group, NC group) and the blank control group reared under normal conditions (normal food group, CK group). At 48, 72 and 96 h, the larvae in the siRNA group were fed a diet containing 2 μg of siRNA, while the NC group was fed a diet containing 2 μg nonsense sequence. The CK group was fed normal food. The samples used for JH titer detecting were collected at 72, 96 and 120 h. The samples used for gene expression determination were collected at 120 h. The collected larval samples were flash-frozen in liquid nitrogen and stored at −80 °C for subsequent qRT-PCR and JH titer detecting. The remaining larvae in the culture plate were transferred to a constant temperature incubator (34 °C, 75% ± 5% relative humidity, darkness) at 144 h until adult emergence. The morphometric characteristics of adult workers were measured.

### 2.3. Inhibition of 15-PGDH in Honey Bee Workers Larvae

To inhibit the expression of *15-PGDH*, a siRNA sequence (sense: 5′-GCU CUC ACC UCG GUA UGU ATT-3′; antisense: 5′-UAC AUA CCG AGG UGA GAG CTT-3′) and a nonsense sequence (sense: 5′-UUC UCC GAA CGU GUC ACG UTT-3′; antisense: 5′-ACG UGA CAC GUU CGG AGA ATT-3′) were synthesized (GenePharma, Shanghai, China). Feeding of siRNA and larvae sampling were implemented as described in Sampling part.

### 2.4. RNA Isolation and Real Time Quantitative PCR

Total RNA was extracted from the samples using the Trizol Up Plus RNA Kit (TransGen Biotech, Beijing, China, ER501-01-V2) following the supplier’s instructions. For mRNA amplification, 1 μg of total RNA from each sample was used to synthesize the first-strand cDNA. The RNA was reverse-transcribed into 20 μL of cDNA using the PrimeScript™ RT Reagent Kit with gDNA Eraser (Perfect Real Time) (TaKaRa, Kusatsu, Shiga Prefecture, Japan, RR047A), following the manufacturer’s instructions. Transcript-specific primer pairs (Table 1) were designed using Oligo 6.0 software and synthesized by Shanghai Sangon Biotech Co., Ltd. (Shanghai, China). The expression levels of *15-PGDH* in the worker bee larval samples were detected by qRT-PCR using the TB Green^®^ Premix Ex Taq™ II (Tli RNaseH Plus) kit (TaKaRa, RR820A), according to the manufacturer’s protocol.

The cDNA samples were serially diluted at 1, 10^−1^, 10^−2^, 10^−3^ and 10^−4^, after which they were subjected to qRT-PCR analysis using the LineGene 9600 Plus Fluorescence Quantitative PCR System (Bioer Technology, Hangzhou, China). The optimal dilution (5×) was then selected for subsequent procedures. The cycling conditions were set as follows: 95 °C for 30 s, followed by 45 cycles of 95 °C for 5 s and 60 °C for 30 s to acquire fluorescence signals, generating amplification and melting curves. This process determined the optimal dilution factor for the samples and validated the designed primers. Standard PCR amplification was performed using cDNA templates to verify the size of amplified fragments. Transcript quantification was then carried out using the TB Green Premix Ex Taq II (TaKaRa, Kusatsu-shi, Japan) on the LineGene 9600 Plus Real-Time PCR System (Bioer Technology). The qRT-PCR reaction system had a total volume of 20 μL, consisting of 1 μL cDNA (200 ng, 1:5 dilution), 0.8 μL forward primer (10 μM), 0.8 μL reverse primer (10 μM), 10 μL TB Green Premix Ex Taq II and 7.4 μL H_2_O. *β-actin* was used as the reference gene [32]. The cycling conditions were 95 °C for 30 s, followed by 45 cycles of 95 °C for 5 s and 60 °C for 30 s to acquire fluorescence signals. The results obtained from qRT-PCR were analyzed using the 2^−ΔΔct^ method based on Ct values. The data were analyzed using one-way ANOVA in SPSS Statistics 27 software, with the TUKEY method employed for multiple comparisons. The final results are expressed as mean ± standard deviation.

### 2.5. Detecting of JH Titer

The samples collected at 72, 96 and 120 h were used for JH titer detecting. Three larvae at 72 h were pooled as one sample. Two larvae at 96 h were pooled as one sample. One larva at 120 h was treated as one sample. In each group, there were three samples and three technical replicates for each sample. The samples were homogenized in phosphate-buffered saline (PBS) at a 1:9 ratio by vortex mixing. Then, the homogenates were centrifuged at 3000 rpm for 20 min, and the supernatant was collected for the next step. JH titer was detected using 10 μL supernatant according to the manufacturer’s instructions of the ELISA kit (Renjie Bio-Technology Co., Ltd., Shanghai, China).

### 2.6. Morphological Measurement of Workers

The workers were sampled immediately after emergence. The emergence weight of the workers was measured using an electronic balance. Then, morphometric measurements of fore wing length (FWL), fore wing width (FWW), femur (FEM), tibia (TIB), basitarsus length (TAL), basitarsus width (TAW), tergite 3 longitudinal (T3), tergite 4 longitudinal (T4), sternite 3 longitudinal (LS3), wax mirror of sternite 3 longitudinal (WML), wax mirror of sternite 3 transversal (WMT), distance between wax mirrors st. 3 (WD), sternite 6 longitudinal (S6L) and sternite 6 transversal (S6T) of body size characteristics were taken from 34 workers, following the method described by Ruttner and Meixner [33,34]. Morphological photographs were captured under consistent and appropriate magnification using a LEICA DMS300 digital microscope (Weztral, Germany). Following image acquisition, the actual size of worker bee morphological features was determined using Adobe Photoshop 2023 software.

### 2.7. Statistical Analyses

In the qRT-PCR result analyses, *β-actin* was used as the reference gene to correct for differences between samples. The relative expression level of the gene was calculated by the 2 ^−ΔΔCt^ method. Specifically, first, the Ct value was calculated between the target gene and the reference gene in each sample (ΔCt = Ct value of the target gene—Ct value of *β-actin*). Then, the ΔΔCt value was calculated by using the ΔCt value of the CK group as the reference benchmark. The relative expression level of the target gene was calculated by the formula 2 ^−ΔΔCt^, which reflects the changing trend of gene expression. In the morphological features analyses, the mean and standard deviation of 14 morphological characteristics (FWL, FWW, FEM, TIB, TAL, TAW, T3, T4, LS3, WML, WMT, WD, S6L, S6T) of worker bees in each group were calculated. The one-way analysis of variance (ANOVA) was used to compare the results among different groups. Tukey’s HSD test was applied to detect significant differences in the mean values of the 14 characteristics between different groups, and the Bonferroni method was used for multiple comparisons to correct the *p*-values.

## 3. Results

### 3.1. The Expression Level of 15-PGDH

**Figure 1:**
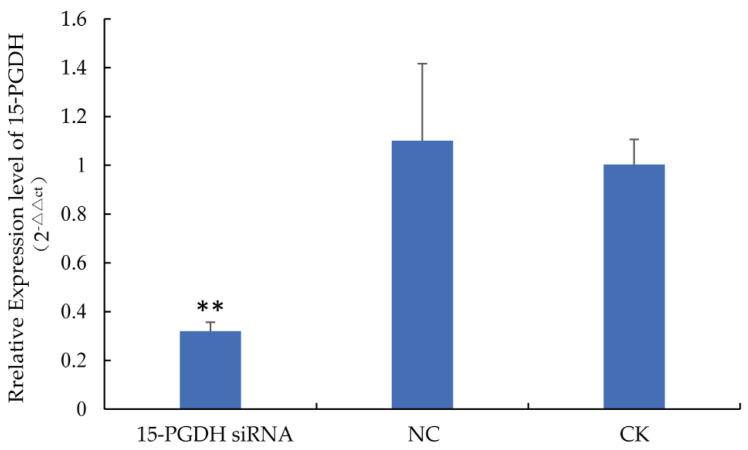
*15-PGDH* expression levels in worker bee larvae at 120 h. *15-PGDH* siRNA and *15-PGDH* siRNA groups. NC, negative control group; CK, blank control group. The expression level of *15-PGDH* in workers of the *15-PGDH* siRNA group was significantly higher than that in the NC and CK groups. Note: ** *p* < 0.01, *n* = 5.

In this experiment, we detected the relative expression level of *15-PGDH* in larvae at 120 h of development (Figure 1). The results show that compared with the CK group, the expression of *15-PGDH* in the siRNA-treated larvae was significantly downregulated (*p* < 0.01), with a silencing efficiency of 70.93%. The qRT-PCR results confirmed the inhibitory effect of this siRNA in worker bee larvae.

### 3.2. JH Titers

**Figure 2:**
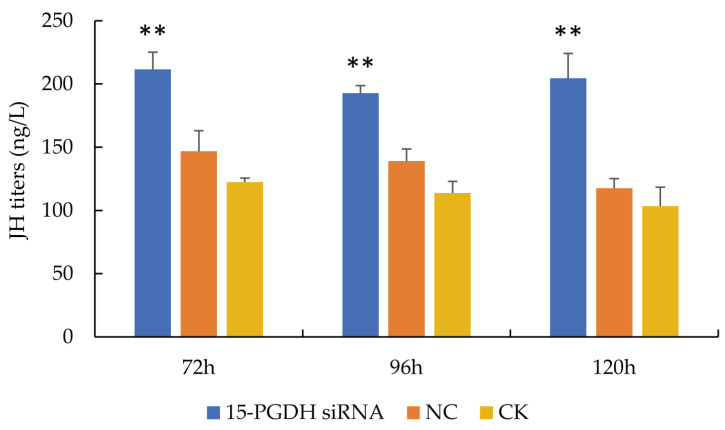
JH titers in worker bee larvae at 72, 96 and 120 h. *15-PGDH* siRNA and *15-PGDH* siRNA groups. NC, negative control group; CK, blank control group. JH titers of worker bees in the *15-PGDH* siRNA group were significantly higher than those in the NC and CK groups at 72, 96 and 120 h. Note: ** *p* < 0.01, *n* = 5.

JH titers in larvae were measured at 72, 96 and 120 h of larval age (Figure 2). The results showed that compared with the CK group, JH titers in the siRNA-treated larvae were significantly increased (*p* < 0.01) at all three stages. These findings suggest that the downregulation of *15-PGDH* expression leads to an increase in JH titers in worker bee larvae.

### 3.3. Morphological Characteristics of Workers

**Figure 3:**
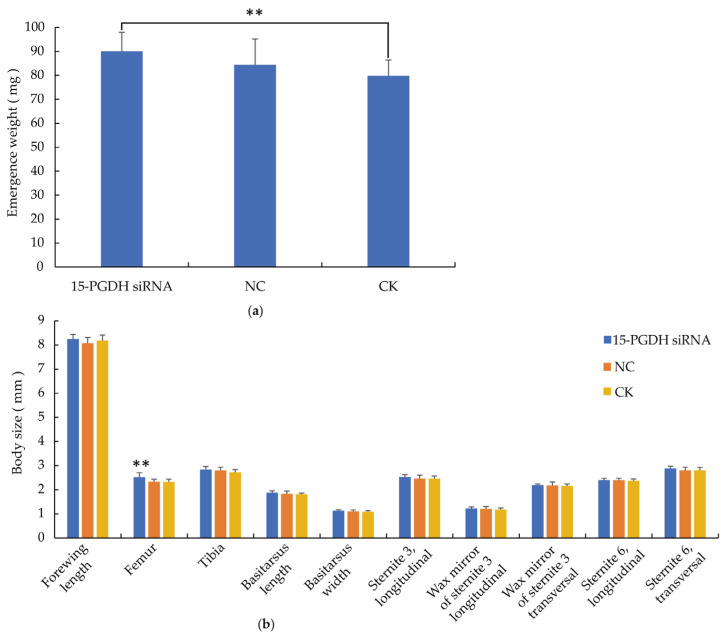
Results of body weight and size. (**a**) Emergence weight of workers. The emergence weight of workers in the *15-PGDH* siRNA group was significantly higher than that in CK group. (**b**) Morphological characteristics of workers. The femur of workers in the *15-PGDH* siRNA group was significantly higher than that in NC and CK group. Note:** *p* < 0.01, *n* = 30.

The results show that the siRNA-treated workers had significantly increased emergence weight (1.13-fold higher than the CK group, *p* < 0.01) and significantly longer FEM (1.08-fold longer than the CK group, *p* < 0.01). Additionally, the siRNA group exhibited increased body size in FWL, TIB, TAL, TAW, LS3, WML, WMT, S6L and S6T compared to the CK group, although the differences were not statistically significant (Figure 3).

## 4. Discussion

As crucial pollinators, honeybees play a pivotal role in maintaining ecological balance [35,36,37]. Healthy and strong colonies exhibit enhanced reproductive capacity and pollination efficiency, thereby improving their productivity of apicultural goods [38]. Moreover, enhanced colony immunity improves resistance to environmental pesticides. As workers constitute the majority of the colony and produce all apicultural products, their health is critically important. Birth body weight, a recognized health parameter, has been identified as a longevity biomarker in honeybees [39]. Worker bees with larger body size exhibit longer lifetime, enhanced foraging and flight capabilities, stronger immune function and higher attack efficiency during colony defense [40,41]. Therefore, promoting worker bees’ body weight and size become an important goal in breeding work in honeybees.

Some important molecular markers for body weight and size have been identified in livestock and poultry. For example, insulin, such as growth factor I (*IGF-I*), was identified associated with emergence weight, body height and body length in pigs [42]. The forkhead box O (*Foxo*) gene affects the growth rate and developmental stages of silkworms by regulating JH degradation and hormone homeostasis, ultimately leading to reduced body size and precocious metamorphosis [43]. *15-PGDH* was the sole catabolic enzyme for prostaglandin E2 (PGE2) [29]. Although no previous studies have demonstrated a role for *15-PGDH* in honeybees, this research reveals that it affects honeybees’ body size development by regulating JH titers. Our results show that suppressing *15-PGDH* expression during critical larval stages (3–5 days of age) significantly increases JH titers and increases the body weight and size of worker bees, including a 1.13-fold increase in body weight (*p* < 0.01) and a 1.08-fold elongation of the femur (*p* < 0.01). The increase in the FEM in bees holds significant biological significance. Studies have reported that the FEM, as an important component of the hind leg, plays a crucial role in supporting the structure of the “pollen basket” on the TIB. A longer FEM can provide more stable support and greater space for the “pollen basket”, which is extremely beneficial for bees to collect and carry pollen, thereby significantly improving their foraging efficiency [44].

These findings are consistent with the known role of JH in honeybees’ larval development. JH maintains larval characteristics in insects, regulates larval development and further influences body size. Increased JH titers during the larval stage prolong the larvae period, allowing for better nutrient accumulation and consequently larger pre-pupal body size [20]. This ultimately results in a larger adult body size and body weight. Previous studies have also demonstrated that increased JH titers may potentially induce worker larvae to develop into queen-like traits [45,46,47]. It is reported that high JH titers promote ovarian cell proliferation in queen larvae [48]. Whether the observed increases in body weight and size in RNAi-treated workers are accompanied by a partial restoration of ovarian function requires further histological investigation in future studies.

Our study found that the suppression of *15-PGDH* expression affects JH titers. *15-PGDH*, a member of the short-chain dehydrogenase/reductase (SDR) superfamily, catalyzes the oxidation of hydroxyl groups (-OH) through hydrogen atom (H) removal (functioning as a catalyst). Given that JH biosynthetic precursors are acidic compounds, we hypothesize that increased *15-PGDH* expression may inhibit JH synthesis. Conversely, suppression of *15-PGDH* reduces its inhibitory effect on JH titers. Increased JH titers further promote increases in body weight and size. Morphometric data showed that *15-PGDH*-suppressed workers exhibited significantly larger FEM compared to controls (*p* < 0.01). Morphological characteristics such as FWL, TIB, TAL and TAW also showed an upward trend. These findings suggest that *15-PGDH* could serve as a molecular marker for body weight and size selection in honeybees’ breeding programs. In this study, we did not observe any adverse effects on the bees resulting from the suppression of *15-PGDH*. Although *15-PGDH* suppression has not been previously reported in insects, studies on human diseases have demonstrated that *15-PGDH* suppression produces target-specific effects without documented adverse consequences [49]. dsRNA is susceptible to enzymatic degradation by nucleases. The dsRNA used in the present study is assumed to degrade within 24 h in larvae. In this study, the siRNA of *15-PGDH* was fed to larvae at 48, 72 and 96 h. It was deduced that the suppression of *15-PGDH* mainly affects larval development. Furthermore, with the advancement of molecular biology, siRNA synthesis techniques have become well-established. The dsRNA was technically designed to reduce non-target effects [50].

The development of honeybees’ larvae is a complex process regulated by a variety of factors [51,52]. Our study found that reducing *15-PGDH* expression in larvae can promote JH titer, leading to increased body weight and size in worker bees. This study identified *15-PGDH* as a molecular marker related to body weight and size in honeybees, which can be used in molecular marker-assisted selection for breeding honeybees. In the future, combining this technology with traditional breeding methods is expected to accelerate the development of honeybees with larger body weight and size. Meanwhile, the finding that *15-PGDH* expression levels modulate JH titer provides novel mechanistic insights into honeybee larval development.

## 5. Conclusions

This study employed RNA interference to silence the *15-PGDH* gene expression in worker larvae of *A. mellifera*, providing the first demonstration of its molecular mechanism in regulating honeybee body size development through modulation of JH titers. The experimental results show that inhibition of *15-PGDH* expression significantly elevates JH titers during the larval stage (*p* < 0.01), leading to a 1.13-fold increase in the body weight of newly emerged workers (*p* < 0.01) and a 1.08-fold elongation of FEM (*p* < 0.01) compared to CK controls, with other morphological indices (e.g., FWL and TIB) also showing upward trends. These findings establish *15-PGDH* as a member of the short-chain dehydrogenase family that negatively regulates JH levels by catalyzing the oxidation of JH biosynthetic precursors, ultimately affecting nutrient accumulation and developmental processes in honeybee larvae. The study not only identifies *15-PGDH* as a key molecular marker for body weight and size traits in honeybees—offering new targets for marker-assisted breeding—but also advances the theoretical framework of JH regulatory networks in insect developmental biology. The future integration of this gene regulation technology with conventional breeding approaches holds promise for accelerating the selection of desirable traits in honeybee populations.

## Figures and Tables

**Table 1:** Primer sequences for qRT-PCR of *15-PGDH*.

Primer Name	Primer Sequence	Ref.
*15-PGDH*-F *15-PGDH*-R	5′-CGGGTTTACCCCATGGTTTC-3′ 5′-CCGTCCCCATAAATCGCTGA-3′	Designed by this study
*β-actin*-F *β-actin*-R	5′-CTGCTGCATCATCCTCAAGC-3′ 5′-GAAAAGAGCCTCGGGACAAC-3′	[32]

## Data Availability

No new data were created or analyzed in this study. Data sharing is not applicable to this article.

## Supplementary Materials

The following supporting information can be downloaded at: https://www.mdpi.com/article/10.3390/life15081230/s1, Table S1: Amount and percentage of diet components in the larval diet.
