# Supplementary material for: Expression of 15-PGDH Regulates Body Weight and Body Size by Targeting JH in Honeybees (Apis mellifera)
Source: Life (Basel). 2025 Aug 3;15(8):1230. doi: 10.3390/life15081230 (PMC12387172; doi:10.3390/life15081230)
Supplement: Supplementary file 1 [file life-15-01230-s001.zip › life-3742433-supplementary.pdf]

Table S1. Amount and percentage of diet components in the larval diet.

Larval age (nth day)	Feed composition					Feed amount
	Royal jelly (%)	Glucose (%)	Fructose (%)	Yeast extract (%)	Sterile water (%)	
1	44.25	5.3	5.3	0.9	44.25	20μl / larvae
2	44.25	5.3	5.3	0.9	44.25	0
3	42.95	6.4	6.4	1.3	42.95	20μl / larvae
4	50	9	9	2	30	30μl / larvae
5	50	9	9	2	30	40μl / larvae
6	50	9	9	2	30	50μl / larvae
